# Free-film small-angle neutron scattering: a novel container-free *in situ* sample environment with minimized H/D exchange

**DOI:** 10.1107/S1600576719000906

**Published:** 2019-02-26

**Authors:** Sebastian W. Krauss, Ralf Schweins, Andreas Magerl, Mirijam Zobel

**Affiliations:** a Chemistry Department, University of Bayreuth, Universitaetsstrasse 30, 95447 Bayreuth, Germany; b Institut Laue–Langevin, DS/LSS, 71 Avenue des Martyrs, Grenoble 38000, France; c Physics Department, Friedrich-Alexander-University Erlangen-Nuremberg, 91052 Erlangen, Germany

**Keywords:** small-angle neutron scattering, SANS, free-film sample environment, hydrogen isotope exchange

## Abstract

The development of a container-free sample environment which is particularly well suited for *in situ* reaction studies of liquid samples by small-angle neutron scattering and related techniques is reported. Hydrogen exchange with the humidity from air is reduced by an encapsulating setup in a bag filled with an inert gas such as He. The effectiveness of this measure is quantitatively accessed by infrared absorption and gravimetry, and further correlated with neutron scattering.

## Abstract 2

A free-film sample environment for neutron scattering, particularly useful for *in situ* small-angle neutron scattering, is presented, providing a sample area of up to 7 × 20 mm and an average thickness of 500 µm. The instrumental background is reduced by 37% compared with standard Hellma cells, and a helium jacketing of the entire setup provides a minimized H/D exchange with the humidity from air.

## 1. Introduction   

Many scattering experiments suffer from insufficient signal, which can be partially hidden by the background, having a significant contribution from the sample environment. Experiments affected include those involving colloidal systems (*e.g.* nanoparticles), soft matter (*i.e.* polymers, micelles) and biological systems, where samples are often present in a highly diluted state. Small-angle X-ray and neutron scattering (SAXS/SANS), specific techniques faced with this problem, are frequently employed for *in situ* studies of nanoparticle formation in order to learn about particle size and shape.

For SAXS, many *in situ* sample environments have been developed, some of them with particular attention devoted to obtaining a low background. This involves stopped-flow devices: to probe fast-reaction kinetics upon mixing, to study the very early process steps in precipitation reactions (Schiener *et al.*, 2015 ▸) or to elucidate shear orientation in microfluidic devices (Taheri *et al.*, 2012 ▸). The equivalents in SANS are a variety of flow-SANS, rheo-SANS or stopped-flow cells (Eberle & Porcar, 2012 ▸; Grillo, 2009 ▸). The role of ligand shells around nanoparticles is relevant to particle formation and stability as the first studies showed (Schindler *et al.*, 2015 ▸; Zobel *et al.*, 2016 ▸; Chatry *et al.*, 1994 ▸; Qu *et al.*, 2011 ▸; Evans *et al.*, 2012 ▸), and the occurrence of co-existing solvation shells with electron densities only marginally different from both the bulk and the ligand layer (Zobel *et al.*, 2015 ▸) makes their observation a challenging task for SAXS. Conversely, SANS can take advantage of the scattering contrast between hydrogen and deuterium; using either deuterated samples or a deuterated solvent, and employing contrast variation and contrast matching, can circumvent some of the above difficulties relating to a weak scattering contrast (Lopez *et al.*, 2018 ▸). However, we are still faced with the challenge of looking at very small scattering contributions of the ligand shell compared with a potentially very large signal coming from the bulk solvent (in particular, for increasing hydrogen content) or the particles. In addition, any container-like sample environment further contributes to the background signal and thus weak scattering contributions may be hard to detect. This is of particular concern when the nanoparticle syntheses are to be studied at low concentrations, which is frequently of interest. Also, the use of a container in nanoparticle experiments runs the risk that the nanoparticles coat the container walls progressively with time and thus observed kinetics and sizes can be distorted. For this reason, SAXS free jets with jet diameters of some 100 µm have been developed. For SANS, levitated-drop setups were shown to work, but limitations resulted from the small drop sizes of 45 µl (Cristiglio *et al.*, 2017 ▸). Both free jets and levitated droplets have the drawback of small sample volumes, whereas in the case of neutron scattering, large beam cross sections of typically 1 × 1 cm are favourable. Here we present a novel container-free sample environment with reduced background scattering, suitable in particular, but not limited, to *in situ* SANS data collection on weakly scattering samples. In the present case, the ‘free film’ features an irradiated sample area of 7 × 10 mm (width × height) and has a thickness of about 500 µm. These parameters can be readily modified for use in different setups.

Because of the open sample environment, hydrogen–deuterium exchange will occur from the humidity of the surrounding air, altering the sample composition over time and thus affecting the desired contrast matching. To solve this issue, we successfully jacketed the setup in a glove bag filled with an inert gas such as He.

## 2. The sample environment   

### 2.1. Technical design   

**Figure 1:**
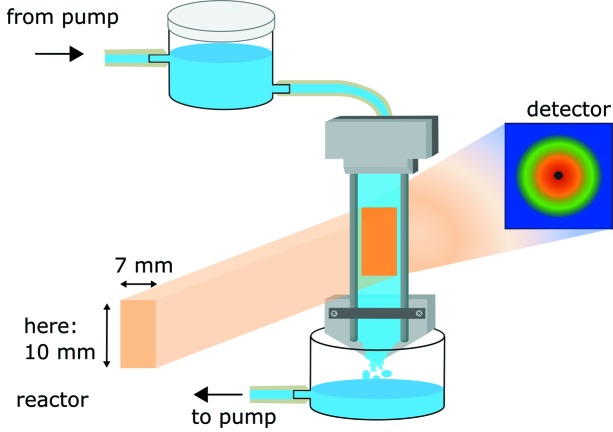
Schematic of the free-film setup. The neutron beam on the free film is indicated by an orange square.

The core of the sample cell consists of two vertical wires kept in place by a metal frame (see scheme in Fig. 1 ▸ and the image of the setup in Fig. S1 of the supporting information). An inlet piece spreads the liquid between the wires and a free-flowing film builds up. This concept, however employing µm-sized wires, has been in use for a while for the generation of THz spectra of water and in the field of optical spectroscopy with films of roughly 3 mm in diameter and ≤100 µm in width (Jin *et al.*, 2017 ▸; Tauber *et al.*, 2003 ▸); here, it is significantly modified to suit the requirements of neutron scattering. We employ 1 mm wires instead, separated by a large distance optimized to 9 mm in the present case. We avoid edge effects close to the wires by optimally aligning the setup by lateral transmission scans in 0.3 mm increments, and this provides a useful sample area of 7 mm multiplied by a variable height of up to 20 mm. A trapezoid-shaped inlet with a two-step dispensing edge made of Teflon was optimized to spread the incoming liquid homogeneously between the wires. An instantaneous film builds up as soon as the flow is initiated. The setup involves a peristaltic pump to circulate the solution between reservoirs at the bottom and the top. The latter is adjusted in height above the free film for optimization of the hydrostatic pressure to control the film initiation and thickness; here, the outlet of the reservoir was 1 cm and the upper liquid level of the reservoir was 5 cm above the inlet to the free film. The upper reservoir could contain up to 115 ml (diameter 5 cm, height 5.9 cm). We used a total volume of 100 ml, including the liquid volume in all the tubing and in the free film, although 30 ml are sufficient for a stable operation. A typical flow rate is 120 ml min^−1^.

### 2.2. H_2_O diffusion from humid air   

To prevent H_2_O introduction into D_2_O dispersions, the entire free-film setup (exposed surface area = 270 mm^2^) is put into a glove bag purged by a constant flow of He gas. The bag was fixed at the neutron guide and the detector tube with holes for the incident beam and scattered beam to avoid scattering from the plastic (Fig. S2). The H/D isotope exchange was periodically monitored (see Section 4.2). As a further fringe benefit, the activation of the gas atmosphere is reduced by this encapsulation.

## 3. Experimental   

### 3.1. Gravimetry   

For the liquid mass/density measurements, an Anton Paar DMA 4500 M density meter with a resolution of 5 × 10^−6^ g cm^−3^ was used. The glass tube was cleaned before every measurement with ethanol and dried in an air stream. All data were taken at a temperature of 293.3 K for 30 s.

### 3.2. IR measurement   

IR measurements were made with an FT/IR-4600 from Jasco, which provides a resolution of 0.7 cm^−1^. The absorption was determined by averaging over 16 scans from which a background run taken before every measurement was subtracted. The measured wavenumbers range from 400 to 8000 cm^−1^ containing 7884 points.

### 3.3. SANS measurement   

SANS measurements were carried out at beamline D11 at the Institut Laue–Langevin (ILL, Grenoble, France) at a wavelength of λ = 4.6 Å and with a sample-to-detector distance of 1.4 m to cover a *Q* range from 0.06 to 0.6 Å^−1^. *Q* = (4π/λ)sinθ is the wavevector transfer. Acquisition times ranged from 2 to 5 min. Careful alignment was carried out to avoid hitting the wires of the free-film rig with the neutron beam. SANS liquid cells from the Hellma QS series made of two 1.25 mm-thick windows of Suprasil quartz and providing a sample thickness of 1 mm were used for reference measurements.

### 3.4. Sample preparation/chemicals   

Heavy water (D_2_O) was purchased from Sigma–Aldrich with an isotopic purity >99.8 at.% D and deionized water was used for light water (H_2_O). For the calibration of IR absorption and gravimetry, mixtures were prepared at 10% H_2_O steps in D_2_O using pipettes. The percentage values refer to volume ratios.

## 4. Results and discussion   

### 4.1. H_2_O content in D_2_O from IR, gravimetry and incoherent scattering   

The following subsections present three independent approaches to determine the H_2_O/D_2_O ratio in water with the objective of providing characterization data for the free-film equipment: IR spectra characterizing molecular vibrations, mass density of the mixture and incoherent scattering.

#### 4.1.1. IR absorption   

The hydrogen-bonded network in liquid water is subject to a continuous rearrangement on the picosecond time scale (Qvist *et al.*, 2011 ▸), involving a temporary dissociation of the water molecules. For this reason, HDO units are formed in H_2_O/D_2_O mixtures. Various spectroscopic techniques such as neutron inelastic scattering (Toukan *et al.*, 1988 ▸) and IR absorption (Maréchal, 1991 ▸; Max & Chapados, 2002 ▸) can provide information on the time-averaged bond probability via the characteristic molecular vibrations in the fluid.

**Figure 2:**
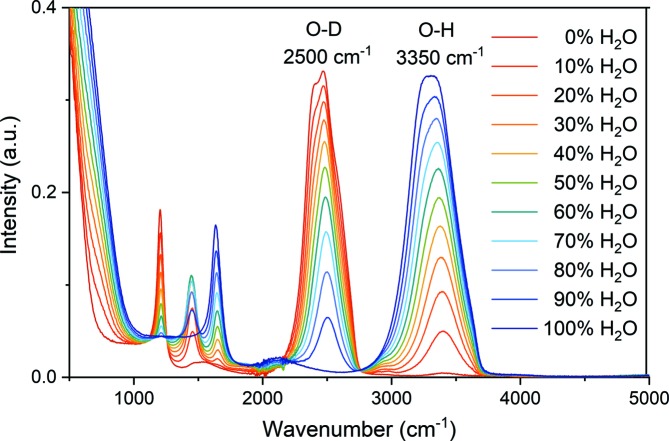
IR absorption spectra for water with different ratios of light and heavy water. The peaks at 2500 and 3350 cm^−1^ relate to molecular vibrations involving O—D and O—H stretching motions, respectively.

**Figure 3:**
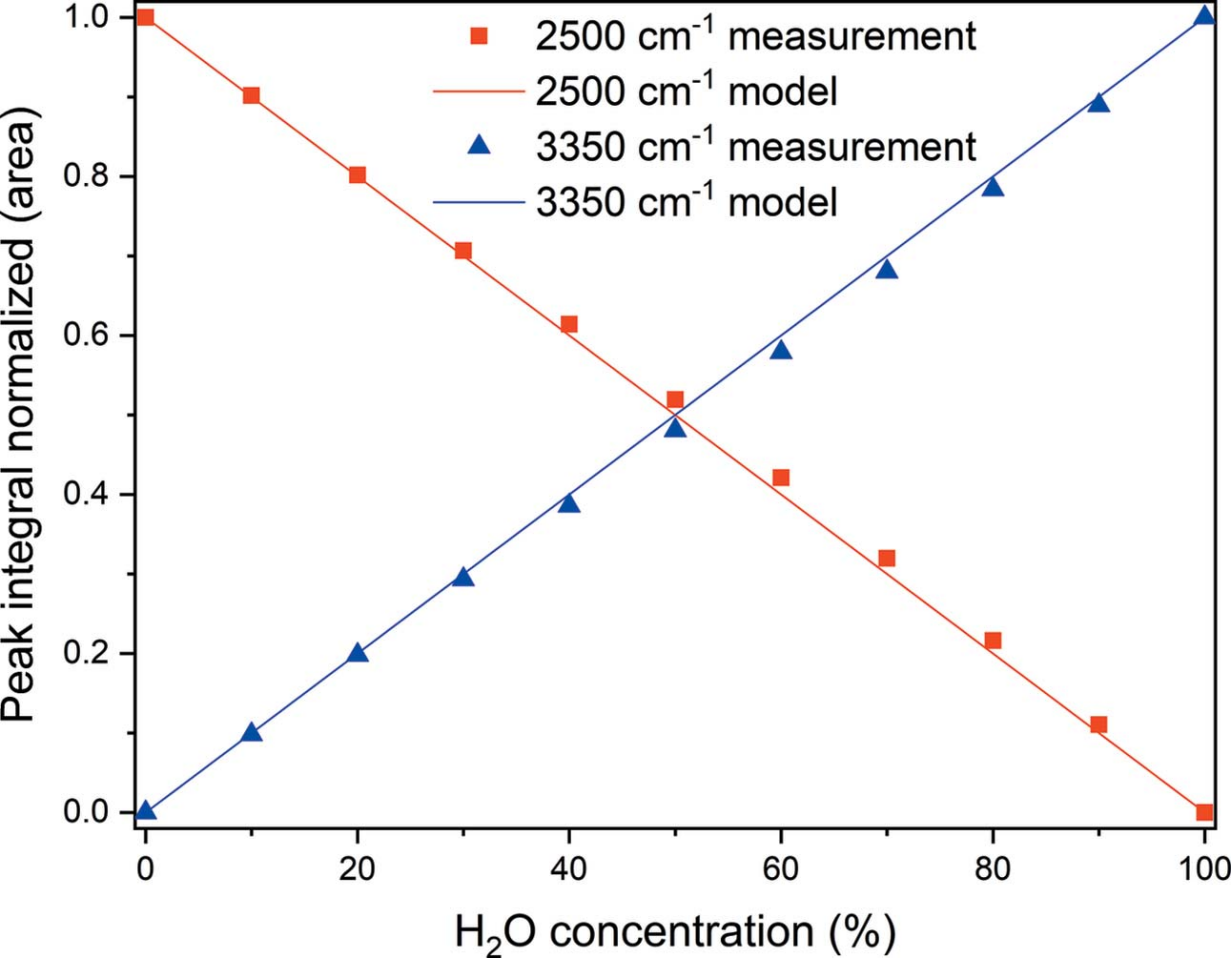
Correlation of H_2_O content with integrated peak areas. The H_2_O concentration on the abscissa is calculated from the volumes of the mixed pure liquids. The experimental data points deviate from the model curve with a standard deviation of 1.5% H_2_O.

Max & Chapados (2002 ▸) identified five principal factors to describe the IR spectra of H_2_O/D_2_O mixtures with varying molar ratios. Herein, the H_2_O content of the H_2_O/D_2_O mixtures was derived in the following way: in the absorption spectra of the different isotope mixtures, two peaks at wavenumbers of 2500 and 3350 cm^−1^ are the most prominent features (Fig. 2 ▸). They are associated with the stretching vibrations of the O—D bond around 2500 cm^−1^ and of the O—H bond around 3350 cm^−1^. On the basis of this knowledge, it suffices for the present to consider these two peaks only. To compensate for the different IR cross sections of the two isotopes, a conversion factor of 1.45 obtained from the peak ratio for the isotope-pure liquids needs to be introduced. Furthermore, for individual runs, the integrated intensities of the peaks have been normalized to the sum of the areas of the peaks at 2500 and 3350 cm^−1^, which represent directly the deuterium and hydrogen concentrations in the mixture, respectively. Fig. 3 ▸ shows the experimental data points for the varying isotopic composition of the two IR peaks together with the calculated model curve. The experimental data points have a standard deviation from the model curve of 1.5% H_2_O, which includes experimental errors. These results demonstrate that IR absorption provides a reliable means to assess the isotopic ratio in aqueous solutions with an accuracy of about 1% H_2_O.

#### 4.1.2. Gravimetry   

**Figure 4:**
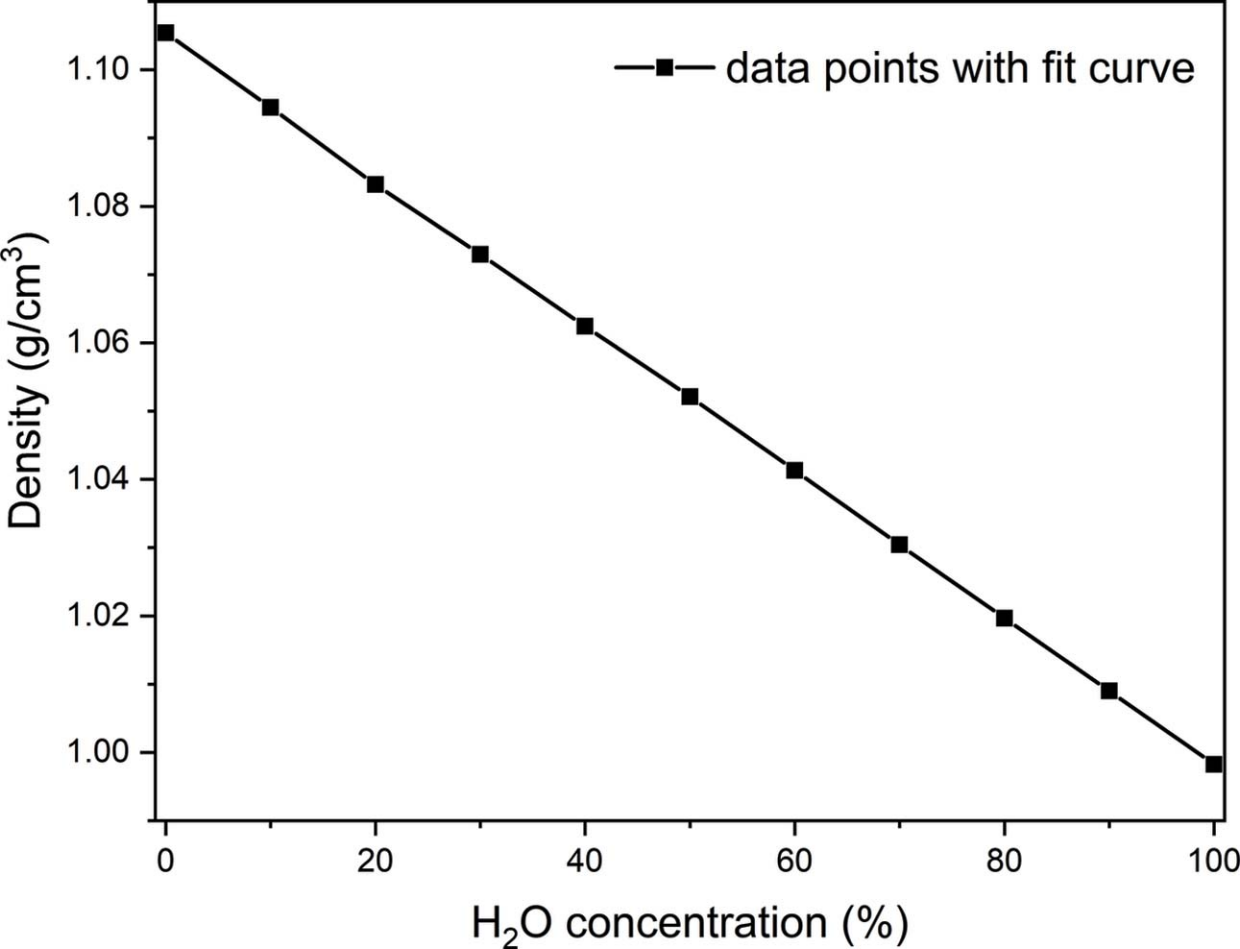
Correlation of the H_2_O concentration with liquid density.

To quantify the isotopic ratio with a further technique, we have undertaken a gravimetric determination. The values for the pure isotopic liquids of 0 and 100% H_2_O from the literature correspond well to our data (Kell, 1967 ▸), and intermediate mixtures are well described by a linear relationship, resulting in an accuracy of 0.024% H_2_O for the H_2_O concentration (Fig. 4 ▸). The accuracy is again the standard deviation of the experimental data points from the linear fit.

#### 4.1.3. Incoherent neutron scattering   

**Figure 5:**
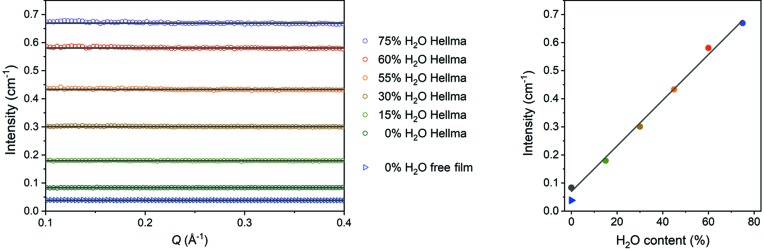
Left panel, upper six curves: data and fits of a constant to the scattering in the flat *Q* regime for different isotopic solutions in 1 mm Hellma cells after subtraction of background scattering from an empty Hellma cell. The lower curve corresponds to the scattering from a free film of pure D_2_O corrected for background from helium, which has been used to determine the film thickness. Right panel: the circles show the incoherent scattering according to the fitted values from the left panel using the same colour code. The triangle at 0% H_2_O shows the value for the free film.

Neutron scattering recorded on the small-angle detector of D11 has been used as a third independent procedure to determine the isotopic ratio of water. To this end, the detected intensities from pure and isotopic water mixtures were corrected for the background taken with an empty Hellma cell and are shown in the left panel of Fig. 5 ▸. In the *Q* region between 0.1 and 0.4 Å^−1^, both heavy and light water are dominated by incoherent scattering described by a constant. The relation of this ‘background’ intensity to the composition of the isotopic mixture is illustrated in the right panel of Fig. 5 ▸. Again, this assessment provides an appropriate means to find the isotopic ratio of hydrogenous solvents like water.

All SANS data are first normalized pixel by pixel before being radially averaged into one-dimensional scattering curves of intensity (cm^−1^) *versus* *Q* (Å^−1^). Data are put on an absolute scale by using the secondary calibration standard H_2_O with 1 mm thickness, having a differential scattering cross section of 0.908 cm^−1^ for 4.6 Å wavelength for D11, as determined by a cross calibration against H/D polymer blends.

In conclusion, we used three independent methods to determine the H_2_O/D_2_O ratios in isotope mixtures; these methods (IR absorption, gravimetry and incoherent scattering) provided different accuracies (see Table 1 ▸). The accuracies were each determined from the standard deviation of their corresponding calibration curve [see Fig. 3 ▸ for IR, Fig. 4 ▸ for gravimetry and Fig. 5 ▸ (right panel) for incoherent scattering].

### 4.2. Reduction of H_2_O exchange by helium glove bag   

**Figure 6:**
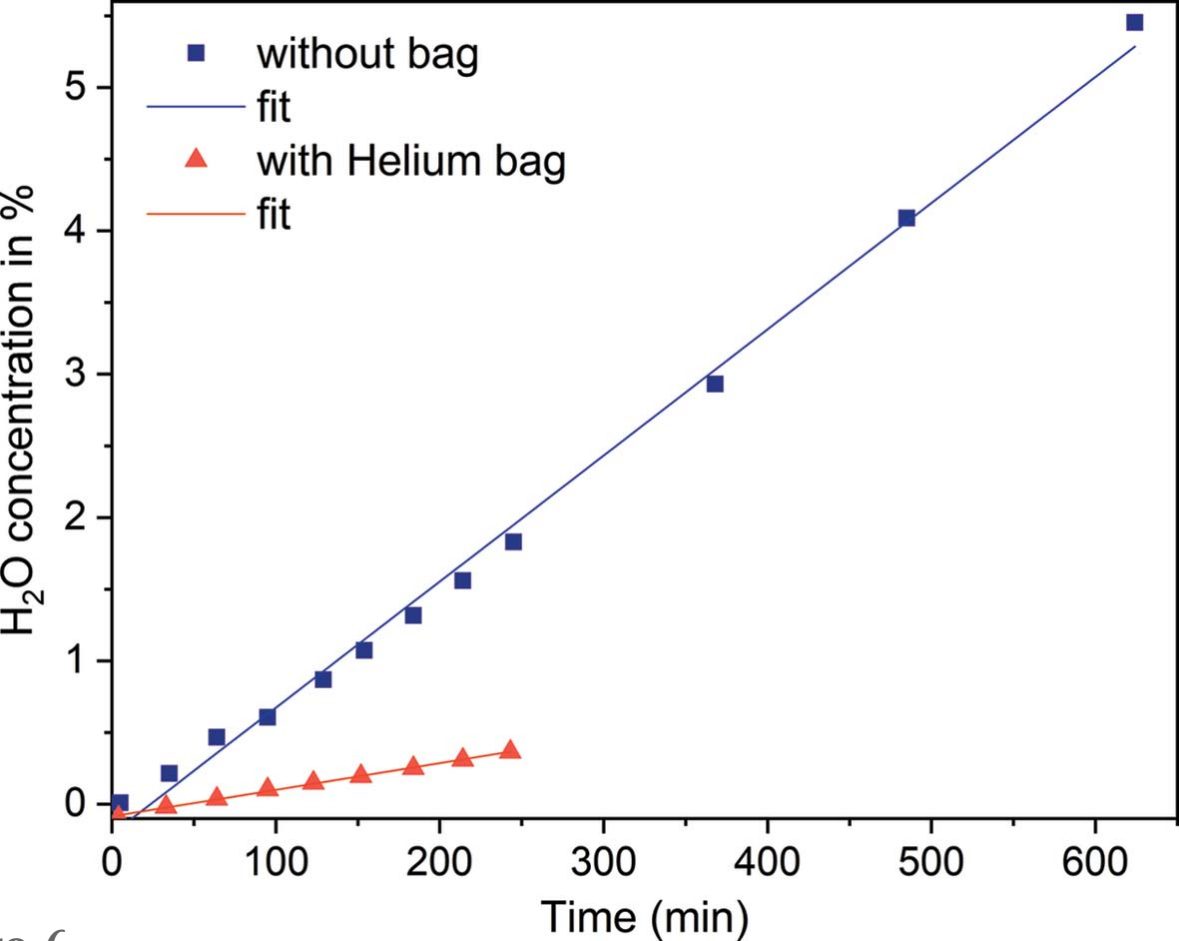
Time dependence of the H_2_O take-up from air by heavy water in a free-film sample cell as determined gravimetrically.

A possible change of the isotopic ratio between light water and heavy water by H_2_O uptake from the humidity of the surrounding atmosphere is of central concern for open sample environments like the one proposed here for a free-film setup. An evolution with time may impede the data analysis significantly as usual procedures for background and other corrections may become doubtful. To this end, we have tested our free-film setup for H_2_O uptake on the SANS diffractometer D11 starting with pure D_2_O. In a first run, the free-film setup with a total circulating liquid volume of 100 ml was put on the sample stage of D11 without further adaptions. The H_2_O uptake was checked periodically by extracting with a syringe 2 cm^3^ of liquid to be analysed gravimetrically. After analysis, this volume was discarded. The data (Fig. 6 ▸) show an increase in the H_2_O concentration of 0.5% per hour. Enveloping the entire free-film setup with a plastic bag, purging it for 5 min with He gas and then maintaining a constant He flow reduces the H_2_O uptake by a factor of five, thus allowing us to collect data even in such open sample conditions with high fidelity.

### 4.3. Performance of the free-film setup   

One distinctive advantage of the free-film setup is that there are no container walls that can become coated during an experiment, particularly when dynamical processes are followed *in situ*. To avoid such features may be very time consuming and complex to correct for.

Evaluating the incoherent scattering from the free film as shown in Fig. 5 ▸ (triangle symbols in blue), we can determine the free-film thickness. Assembling and running the setup twice from scratch, the thicknesses for the two runs are 0.43 ± 0.01 and 0.55 ± 0.01 mm. The difference in the film thickness can be explained by a small change in the hydrostatic pressure on the film inlet (relative height of upper reservoir to free film, liquid volume in upper reservoir). To determine the thickness of the pure D_2_O film the ratio of the incoherent scattering from the film (triangles in Fig. 5 ▸) and of pure D_2_O in the Hellma cell (black circles) was calculated. As the Hellma cell has a thickness of 1 mm, the calculated ratio provides the film thickness according to equation (1):
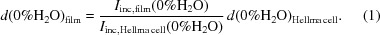



The most notable feature of the free-film setup concerns the background conditions in SANS measurements. From the count rate of the detector at various distances (Table 2 ▸), it appears that the instrumental background is reduced on average by 37% when changing from an empty Hellma cell to the free-film setup. This holds for both a setup in air and one under an He atmosphere as provided by the encasing with an He-filled bag.

## 5. Conclusions   

We have developed a novel free-film setup for neutron scattering, particularly well suited (but not limited) to SANS measurements on liquids, which is container free in the region of the neutron beam. It provides an average film thickness of 0.49 mm and a sample area with a width of 7 mm and a height of up to 20 mm. Tests on D11 at the ILL have shown that the instrumental background is reduced by 37% compared with standard Hellma cells. We further show that helium jacketing strongly reduces H/D exchange from air with the free film. The hydrogen contamination was efficiently determined by gravimetry, IR spectroscopy and incoherent scattering, with gravimetry being the most precise technique; however, IR as a more readily available technique also provides reliable information.

The ILL data are accessible at http://doi.ill.fr/10.5291/ILL-DATA.1-10-38.

## Supplementary Material

Supporting information file. DOI: 10.1107/S1600576719000906/vg5102sup1.pdf

## Figures and Tables

**Table 1:** Accuracy in determining the H_2_O concentration as determined from IR, gravimetry and incoherent scattering

Method	IR absorption	Gravimetry	Incoherent scattering
Standard deviation	1.5% H_2_O	0.024% H_2_O	1.4% H_2_O

**Table 2:** Background reduction of free-film setup compared with Hellma cells for three different detector distances

Detector distance (m)	Detector count rate (counts s^−1^): empty Hellma cell	Detector count rate (counts s^−1^): free film	Background reduction (%)
1.4	42871	26930	−37
5	2343	1386	−41
13	108	71	−34
