# Supplementary material for: Free-film small-angle neutron scattering: a novel container-free in situ sample environment with minimized H/D exchange
Source: J Appl Crystallogr. 2019 Feb 26;52(Pt 2):284–8. doi: 10.1107/S1600576719000906 (PMC6448684; doi:10.1107/S1600576719000906)
Supplement: Supplementary file 1 [file j-52-00284-sup1.pdf]

JOURNAL OF APPLIED CRYSTALLOGRAPHY

Volume 52 (2019)

Supporting information for article:

Free-film SANS – a novel container-free *in situ* sample environment with minimized H/D-exchange

Sebastian W. Krauss, Ralf Schweins, Andreas Magerl and Mirijam Zobel

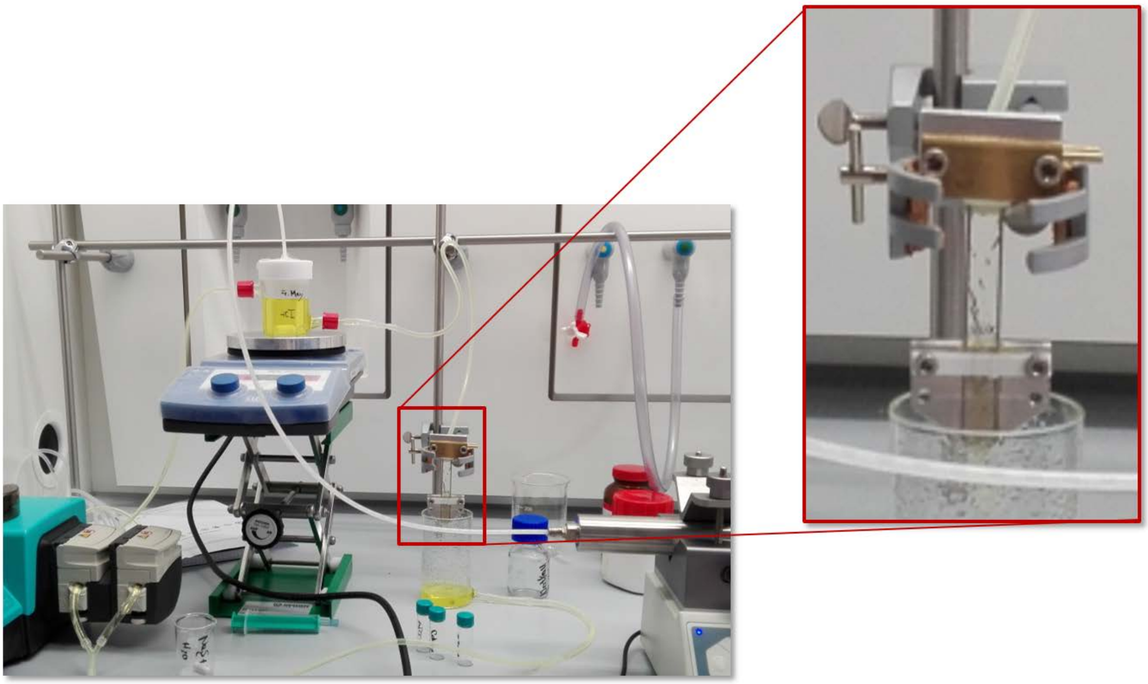

Figure S1 **Figure S1** Photograph of the free-film setup in the laboratory, pumping an aqueous CdS nanoparticle suspension for better visibility of the free-film between the wires in the enlarged area.

Figure S2

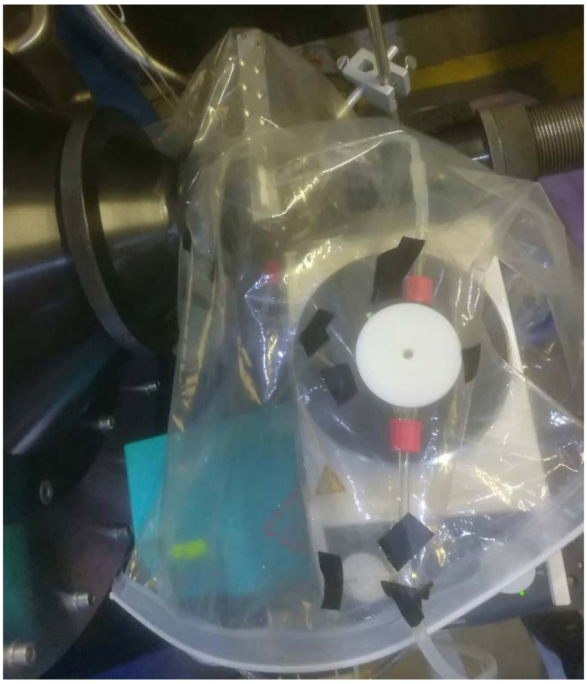

Figure S3

Figure S2 Photograph of the free-film setup at beamline D11 with the Helium bag (top-view)

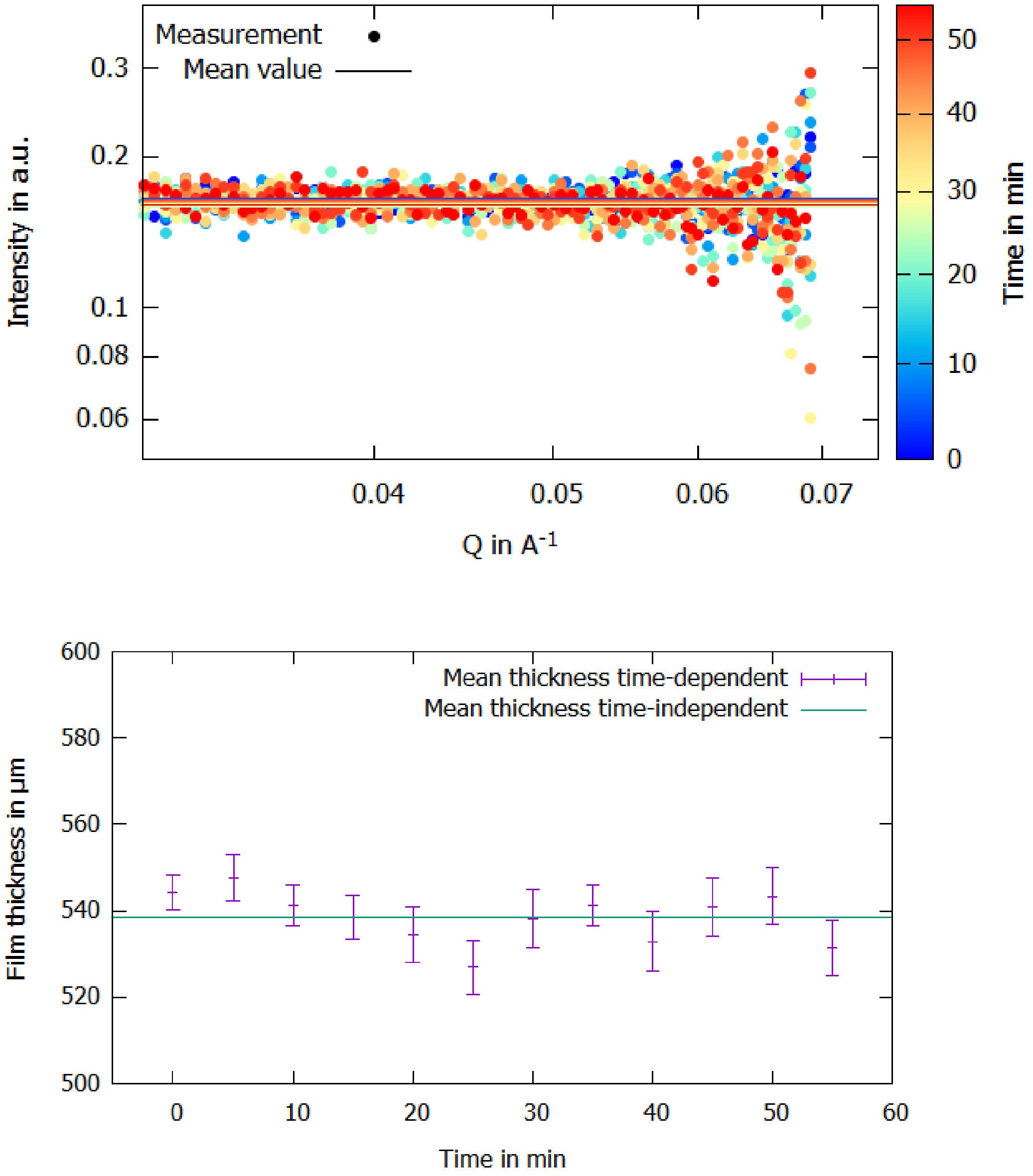

Figure S3 Top: SANS data of D70 for a detector distance of 13 m over a time of 55 min in 5 min intervals together with constants fitted to the individual data sets for an isotopic mixture with H/D = 3/7. Bottom: Film thicknesses as determined from equation (1) showing that the film thickness only slightly fluctuates around the average value.

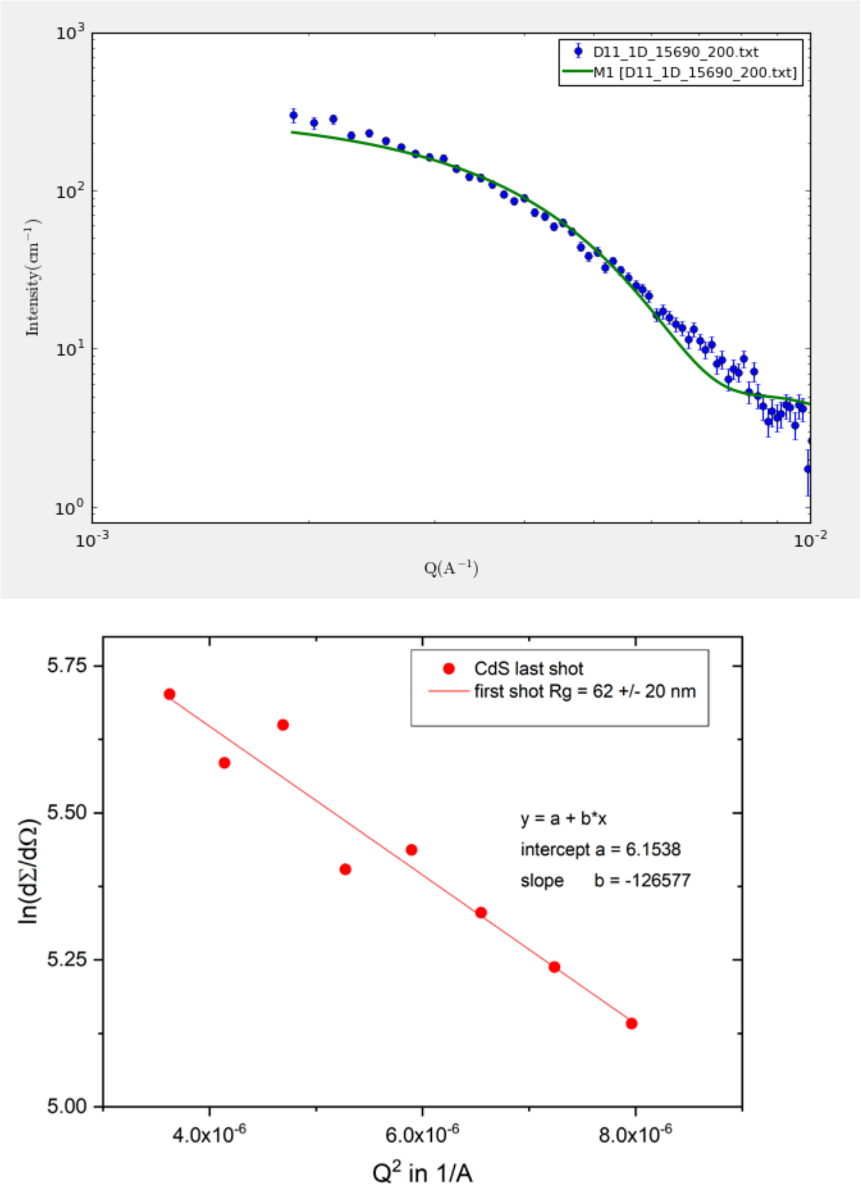

Figure S4 SANS data of EDTA-stabilized CdS nanoparticles in water during the in-situ particle formation of CdS nanoparticles after a reaction time of 190 min in aqueous solution from $CdCl_2$ and Na_2S in presence of EDTA (ethylenediaminetetraacetic acid), all equimolar [Cd^{2+}] = [S^{2-}] = [EDTA]. Top: The solid line represents a fit with a spherical shape factor (preliminary) yielding a radius of 66.2 nm +/- 0.6 nm. Bottom: Guinier fit resulting in Guinier radius of 62 +/- 20 nm.
